# The effects of adolescents' sleep duration on life satisfaction: utilizing the autoregressive cross-lagged (ARCL) model

**DOI:** 10.3389/frsle.2024.1483543

**Published:** 2025-01-29

**Authors:** Eun Jin Jung

**Affiliations:** Center for Regional and Industrial HRD Research, Korea Research Institute for Vocational Education and Training, Sejong, Republic of Korea

**Keywords:** adolescents, sleep duration, life satisfaction, autoregressive cross-lagged (ARCL) model, Korean Children and Youth Panel Survey (KCYPS)

## Abstract

**Introduction:**

Sleep duration in youth is a key factor that significantly influences mental health. However, there have been limited studies examining the direct impact of adolescents' sleep on life satisfaction. This study aims to analyze the effects of adolescents' sleep duration on their life satisfaction.

**Methods:**

For this purpose, data from the Korean Children and Youth Panel Survey (KCYPS) conducted by the National Youth Policy Institute (NYPI) were utilized, specifically focusing on the 4th-grade panel data (N = 2,378; 52.4% male; 47.6% female). Analysis revealed a rapid decline in youth sleep duration upon entering junior high school. Consequently, the data were categorized into two periods for analysis: Year I (4th grade) to Year IV (7th grade), and Year IV (7th grade) to Year VII (10th grade).

**Results:**

Using the autoregressive cross-lagged (ARCL) model, it was found that sleep duration during Years IV-VII significantly influenced life satisfaction over time. An increase in sleep duration led to a subsequent increase in life satisfaction over time with statistical significance. Specifically, sleep duration during 7th grade exhibited a significant static influence on life satisfaction at 8th grade (β = 0.025, *p* < 0.03), while sleep duration during 8th grade statically affected life satisfaction during 9th grade (β = 0.026, *p* < 0.03). Moreover, sleep duration at 9th grade had a static influence on life satisfaction during 10th grade, with statistical significance (β = 0.027, *p* < 0.03).

**Discussion:**

Based on the findings of this study, implications and suggestions for future research were discussed. It is essential to create an environment that promotes adequate sleep for adolescents, even after they enter junior high school.

## 1 Introduction

Life satisfaction refers to an individual's personal judgment of their quality of life across various domains based on their own criteria (Fujita and Diener, 2005). Multiple factors influence adolescents' life satisfaction. Key factors include environmental aspects such as school life and satisfaction with academic performance, as well as personal characteristics like ego-resilience and leisure satisfaction. Specifically, school life (Son et al., 2017), peer attachment and ego-resilience (Yoo et al., 2005), leisure satisfaction (Cho, 2004), and satisfaction with academic grades (Kim O. et al., 2006) play critical roles in enhancing the life satisfaction of Korean youth. Even though the factors affecting adolescents' life satisfaction have been identified, the percentage of Korean youth with high life satisfaction remains relatively low.

According to an analysis of statistics on Korean youth aged 13–24 in 2016, 46.2% reported being stressed about their overall life (Statistics Korea & Ministry of Gender Equality Family, 2017). When the causes of death among youth aged 7–24 were analyzed for 2015, “intentional self-harm” was the leading cause, followed by “transport accidents” and “malignant neoplasm (cancer).” This ranking remained unchanged from 2007 to 2015. In relation to this issue, the Ministry of Health and Welfare (2011) stated that insufficient sleep in adolescents increases the likelihood of smoking, drinking, and suicide. Practically, recent studies have indirectly suggested that sleep disturbances or problem in adolescents may influence their depression and anxiety (Chai and Bian, 2024; Kirschbaum-Lesch et al., 2021).

In more detail, based on data from the 9th Youth Health Behavior Online Survey (2013), Park (2015) found that among students who slept less than 5 hours a day, 22.4% had thought about killing themselves. In contrast, among students who slept more than 8 hours a day, only 12.9% had suicidal thoughts. The rate of suicidal thoughts was twice as high among students with sleep deprivation compared to those who had enough sleep. Furthermore, according to the findings of a meta-analysis by Baldini et al. (2024), adolescents with sleep disturbances are more likely to attempt suicide and experience suicidal ideation compared to the control group. This study conducted a meta-analysis based on 19 studies from nine countries, comprising 628,525 adolescents with sleep disturbances and 567,746 in the control group.

As stated above, the sleep duration of youth is a major factor significantly influencing mental health. However, there have been few studies analyzing the direct impact of adolescents' sleep on life satisfaction, particularly among Korean youth. A recent study (Moon et al., 2023) utilized raw data from the 2021 Community Health Survey in Korea to analyze the relationship between sleep duration and subjective health levels. The survey targeted adults aged 19 and older residing in sample households at the time of the survey, with a total of 141,524 participants included in the analysis. The results indicated that sleeping between 7 and 9 h was associated with the highest subjective health levels, while sleeping more than 9 h was linked to a decrease in subjective health. Additionally, a recent study (Kudrnáčová and Kudrnáč, 2023) conducted in the Czech Republic analyzed data from the Czech Household Panel Study (2018–2020), and found that sleep quality was the most significant predictor of the quality of life indicators. However, despite such prior research, it is still difficult to confirm the direct influence of sleep duration on life satisfaction of Korean adolescents. Thus, this study aims to investigate the direct influence of sleep duration on life satisfaction in Korean teenagers using panel data.

Thus, the research questions in this study are as follows:

Q1. Does the duration of adolescents' sleep affect their life satisfaction over time?

To investigate this research question, this study^1^ utilized 4th-grade panel data from the Korean Children and Youth Panel Survey (KCYPS) provided by the National Youth Policy Institute (NYPI). Established in 1989, NYPI has conducted research on various policies related to Korean youth. KCYPS collected data from 2010 to 2016. The data from a total of 7 years (2010–2016) were used and analyzed. Upon examining adolescents' average sleep duration, it was found that Korean teenagers' sleep duration rapidly decreased as soon as they entered junior high school (at age 14). During this time, they suffer from sleep deprivation due to intense studying aimed at entering prestigious universities. Therefore, the data were divided into two parts: the first part is before entering junior high school, and the second part is after entering junior high school.

More specifically, this study aimed to investigate whether sleep duration during the 4th−7th grades (effects of sleep duration during the 4th−6th grades on life satisfaction during the 5th−7th grades) and 7th−10th grades (effects of sleep duration during the 7th−9th grades on life satisfaction during the 8th−10th grades) significantly influences life satisfaction. To determine causality among the variables over time, the autoregressive cross-lagged (ARCL) model was used.

### 1.1 Sleep and life satisfaction

Sleep and life satisfaction are closely related. From a qualitative perspective, poor sleep during adolescence increases the risk of depression (Danielsson et al., 2013). Danielsson et al. (2013) explained that poor sleep quality can lead to catastrophic worry and worsen depressive symptoms. According to a survey tracking 1,760 high school students aged 16–18 in Sweden, conducted over 3 years from 2006 to 2008, poor sleep quality influenced catastrophic worry and aggravated the severity of depression. Catastrophic worry refers to the act of continuously having negative thoughts about future events, such as “What should I do if something happens?” (Startup and Davey, 2001). This can be understood through the “cognitive vulnerability-stress paradigm” (Danielsson et al., 2013), which is the opposite of the “diathesis-stress paradigm.” The diathesis-stress paradigm suggests that the degree of stress is inherent, whereas the cognitive vulnerability-stress paradigm argues that stress is formed by interactions between personal predispositions and environmental factors (Riskind and Alloy, 2006). In other words, as catastrophic worry becomes more serious, depressive symptoms also worsen (Garnefski et al., 2002). Steptoe et al. (2008) also discovered that positive emotions and subjective wellbeing were negatively related to sleep problems in their study of individuals aged 58–72. As mentioned earlier, good sleep is critical for maintaining positive emotions.

Moreover, according to previous research (Shin and Kim, 2018), sleep disorders generally lead to a scarcity of cognitive resources. This scarcity would prompt individuals to perceive the world competitively, ultimately resulting in zero-sum beliefs. Zero-sum beliefs are based on the assumption that there is a finite amount of goods in the world (Von Neumann and Morgenstern, 1944), and individuals with high levels of these beliefs tend to perceive happiness as fixed. Sleep plays a role in replenishing depleted resources in humans (Saper et al., 2005). Thus, when sleep is lacking, people typically perceive the world as lacking in resources, which leads them to understand happiness in a zero-sum manner. Ultimately, sleep deprivation could temporarily reduce life satisfaction. Based on this logic, empirical evidence has demonstrated that poor sleep quality decreases life satisfaction (Shin and Kim, 2018).

From the perspective of sleep duration, sufficient sleep duration also positively influences emotion regulation ability (Yoo et al., 2007). Yoo et al. (2007) divided the subjects into two groups: one with sufficient sleep and one with sleep deprivation, and conducted an experiment on young, healthy adults aged 18–30. When negative pictures were presented to both groups, the amygdala response was significantly heightened in the sleep-deprived group, with the duration of this heightened response being longer. The amygdala plays a key role in processing information related to motivation, learning, and emotion. Furthermore, Fredriksen et al. (2004) revealed that as sleep duration decreases, depression increases, according to their study conducted among junior high school students in Chicago, USA. In summary, in the case of sleep deprivation, there is a high likelihood of reacting sensitively to negative incidents or situations, and this sensitivity could persist for a prolonged period.

### 1.2 Sleep duration of Korean youth

As mentioned above, the quality and duration of sleep are closely related to personal emotions. However, studies on this matter have mostly been conducted on Western populations. In the Republic of Korea, it is still challenging to find research on the relationship between sleep and personal emotions. Thus, this study aimed to investigate whether adolescents' sleep duration has a significant influence on their life satisfaction, using the 4th-grade panel data from NYPI's KCYPS. The annual changes in average youth sleep duration were examined and are presented in Table 1.

**Table 1:** Sleep duration on school day (4th grade panel; unit: h:min).

	**Year**							
	**Year I Age 11**	**Year II Age 12**	**Year III Age 13**	**Year IV Age 14**	**Year V Age 15**	**Year VI Age 16**	**Year VII Age 17**	**Overall mean (Sum)**
Sleep duration	8:55	8:46	8:26	7:43	7:32	7:17	6:20	
Time difference		−9	−20	−43	−11	−15	−57	−25.83 (−155)

As indicated in Table 1, when the 4th graders (age 11) entered high school, sleep duration decreased by ~155 min over 7 years. In other words, since the average sleep duration at age 11 was 535 min (8 h and 55 min), it declined by 28.9%. Particularly noteworthy is the significant drop in youth sleep duration at ages 14 (entering junior high school) and 17 (entering high school). This suggests that as students progress to higher levels of education, they face increased academic demands, which leads to sleep deprivation. According to the recommended sleep duration by age as announced by the National Sleep Foundation (NSF), the recommended sleep duration is 9–11 h for grades 4 through 7, and 8–10 h for grades 8 through 10. However, Korean adolescents' sleep duration is notably lower than the recommended level (Ha, 2017).

In terms of the decrease in sleep duration per year, the following results were obtained: 9 min in Years I–II (4th−5th grades), 20 min in Years II–III (5th−6th grades), 43 min in Years III–IV (6th−7th grades), 11 min in Years IV–V (7th−8th grades), 15 min in Years V–VI (8th−9th grades), and 57 min in Years VI–VII (9th−10th grades). The average decrease in sleep duration was 25.83 min.

A rapid decline was observed in Years III–IV (43 min) and VI–VII (57 min). Thus, this study investigated whether sleep duration had a significant influence on life satisfaction during the first half (4th−7th grades: the effects of sleep duration of 4th−6th graders on the life satisfaction of 5th−7th graders) and second half (7th−10th grades: the effects of sleep duration of 7th−9th graders on the life satisfaction of 8th−10th graders) of adolescence.

## 2 Materials and methods

### 2.1 Participants

This study utilized the 4th-grade panel data from the KCYPS. Data collection was conducted with the consent of parents, and no specific exclusion criteria were applied. The 4th-grade panel data were collected over 7 years, starting from the cohort of 4th graders in 2010. Specifically, the subjects were 4th graders in 2010, 5th graders in 2011, 6th graders in 2012, 7th graders in 2013, 8th graders in 2014, 9th graders in 2015, and 10th graders in 2016. In 2010, the number of respondents targeted for the 4th-grade panel survey was 2,378. Among them, 52.4% were male, while 47.6% were female. The number of respondents in 2011 was 2,264, followed by 2,219 in 2012, 2,092 in 2013, 2,070 in 2014, 2,061 in 2015, and 1,977 in 2016. The retention rate of the original sample in 2016 was recorded at 83.1% (Male: 52.3%, Female: 47.7%).

### 2.2 Measures

#### 2.2.1 Sleep duration

To measure sleep duration, respondents were asked the question, “What time do you usually go to bed and wake up in the morning?” under the category of “This question is about how you spend a day during this period.” Then, they were instructed to answer as follows: “I go to bed at_**:_** and wake up at **_:_** on weekdays (Monday–Friday).” Students' sleep duration was calculated from each response and converted into minutes for data analysis. The average sleep duration was as follows: 8 h and 55 min in Year I, 8 h and 46 min in Year II, 8 h and 26 min in Year III, 7 h and 43 min in Year IV, 7 h and 32 min in Year V, 7 h and 17 min in Year VI, and 6 h and 20 min in Year VII.

#### 2.2.2 Life satisfaction

To measure life satisfaction, respondents were asked to answer three questions on a four-point scale (1: Absolutely to 4: Absolutely Not) under the category of “This part is about how you think of your life. Please choose the best answer in each of the following questions.” The three questions were: “I enjoy my life,” “I don't have much worry,” “I think I live a happy life.” For analysis, each question was reverse-scored using the scale designed by Kim S. et al. (2006). Regarding the reliability of the three questions by year, the results were as follows: 0.80 in Year I, 0.81 in Year II, 0.86 in Year III, 0.84 in Year IV, 0.81 in Year V, 0.82 in Year VI, 0.81 in Year VII. Then, the mean was used for analysis.

#### 2.2.3 Control variables

In terms of control variables, personal health conditions and gender were considered. Gender was included as a control variable because life satisfaction can vary by gender (Montepare and Lachman, 1989). Additionally, health conditions were included because personally perceived health conditions are closely related to happiness (Palmore and Luikart, 1972). To measure health conditions, respondents were asked the question “How healthy do you think you are compared to your peers?” and were instructed to answer using a four-point scale (1: Very Healthy, 2: Healthy, 3: Not Healthy, 4: Very Unhealthy). The analysis was divided into two parts: Years I–IV analysis and Years IV–VII analysis. For each analysis, health conditions in the 1st and 4th years were considered as control variables.

Health conditions in Year I showed significant correlations (*r* = −0.234, −0.188, −0.170, −0.140, *p* < 0.001) with life satisfaction during Years I–IV. Similarly, health conditions in Year IV were correlated with life satisfaction during Years IV–VII with statistical significance (*r* = −0.284, −0.210, −0.216, −0.163, *p* < 0.001). A score close to “1” indicates that respondents perceive themselves as healthy. Therefore, negative correlations with life satisfaction suggest that as health conditions improve, life satisfaction increases. These results align with findings from previous studies.

### 2.3 Data analyses

For analysis, the Autoregressive Cross-Lagged (ARCL) model was adopted. This model allows for the estimation of cross-lagged effects between variables (Hong et al., 2007) and verification of autoregressive coefficients. Moreover, it has the advantage of assessing causality (Ryu and Seo, 2015; Park and Lee, 2013) by considering the temporal precedence of variables (Kim et al., 2009).

In this model, a value at time [*t*] can be predicted based on measurements at time [*t*−1], known as the “autoregressive coefficient.” Additionally, the prediction of the value of another variable at time [*t*] based on measurements at time [*t*−1] is defined as the “cross-lagged coefficient.” Therefore, a value at time [*t*+1] must be predicted using the value at time [*t*]; it cannot be predicted solely from measurements at time [*t*−1] (Curran and Bollen, 2001).


$$\begin{array}{l} y_{i}\left[t\right]=\beta_{0}\left[t\right]+\beta_{i}y_{i}\left[t-1\right]+\beta_{2}Z_{i}\left[t-1\right]+e_{i}\left[t\right] \end{array}$$

In the ARCL model, variables can be designated as either latent or observed (Park and Lee, 2013). Hence, this study assessed the reliability of life satisfaction and analyzed its mean by year using AMOS. Additionally, to address missing values, full information maximum likelihood (FIML) was employed (Hong and Yoo, 2004). FIML is advantageous as it provides more accurate estimates of parameters (Arbuckle, 1996).

To evaluate the model, root mean square error of approximation (RMSEA), normed fit index (NFI), and comparative fit index (CFI) were utilized as fit indices. Because the chi-square test is sensitive to sample size (Hong and Yoo, 2004), fit indices were preferred. A goodness-of-fit is considered satisfactory if the NFI and CFI are 0.90 or higher, and the RMSEA is 0.08 or less (Browne and Cudeck, 1993).

## 3 Results

### 3.1 Descriptive statistics

The descriptive statistics and correlation analysis results are presented in Table 2. The findings indicate that the absolute value of kurtosis did not exceed “7,” and the skewness was less than “2.” Hence, it is reasonable to conclude that the variables exhibit normality (Kline, 2010).

**Table 2:** Correlations of variables.

			**Sleep duration on school day**							**Life satisfaction**							**Control variable**		
																	**Gender**	**Health condition check**	
			**Year I**	**Year II**	**Year III**	**Year IV**	**Year V**	**Year VI**	**Year VII**	**Year I**	**Year II**	**Year III**	**Year IV**	**Year V**	**Year VI**	**Year VII**		**Year I**	**Year IV**
Sleep duration on school day		Year I	1															
		Year II	0.67^**^	1														
		Year III	0.33^**^	0.39^**^	1													
		Year IV	0.19^**^	0.26^**^	0.35^**^	1												
		Year V	0.17^**^	0.25^**^	0.29^**^	0.39^**^	1											
		Year VI	0.15^**^	0.21^**^	0.28^**^	0.33^**^	0.45^**^	1										
		Year VII	0.07^**^	0.13^**^	0.11^**^	0.18^**^	0.28^**^	0.36^**^	1									
Life satisfaction		Year I	0.00	0.00	0.00	0.01	0.02	−0.01	−0.04^*^	1								
		Year II	0.05^*^	0.03	0.01	0.06^**^	0.07^**^	0.01	−0.01	0.38^**^	1							
		Year III	0.03	0.01	0.08^**^	0.11^**^	0.11^**^	0.09^**^	0.06^*^	0.29^**^	0.40^**^	1						
		Year IV	0.02	0.02	0.06^*^	0.10^**^	0.10^**^	0.08^**^	0.02	0.25^**^	0.31^**^	0.45^**^	1					
		Year V	0.06^*^	0.05^*^	0.06^**^	0.07^**^	0.11^**^	0.08^**^	−0.01	0.22^**^	0.30^**^	0.38^**^	0.46^**^	1				
		Year VI	0.02	−0.01	0.04	0.04	0.08^**^	0.08^**^	0.03	0.21^**^	0.26^**^	0.34^**^	0.41^**^	0.46^**^	1			
		Year VII	0.02	0.04	0.04	0.06^**^	0.07^**^	0.08^**^	0.05^*^	0.16^**^	0.21^**^	0.26^**^	0.35^**^	0.41^**^	0.47^**^	1		
Control variable	Gender		0.07^**^	0.06^**^	0.15^**^	0.18^**^	0.19^**^	0.23^**^	0.14^**^	0.00	0.08^**^	0.19^**^	0.22^**^	0.15^**^	0.14^**^	0.15^**^	1	
	Health condition check	Year I	−0.01	0.02	0.03	−0.02	−0.02	0.02	0.01	−0.23^**^	−0.19^**^	−0.17^**^	−0.14^**^	−0.12^**^	−0.12^**^	−0.13^**^	0.04	1	
		Year IV	−0.02	−0.01	−0.01	−0.01	−0.01	0.02	−0.01	−0.14^**^	−0.15^**^	−0.24^**^	−0.28^**^	−0.21^**^	−0.22^**^	−0.16^**^	0.08^**^	−0.27^**^	1
M			8:55	8:46	8:26	7:43	7:32	7:17	6:20	3.21	3.26	3.23	3.15	3.03	3.06	2.97	1.47	1.70	1.74
SD			52.24	50.57	52.33	58.10	61.27	62.78	67.10	0.70	0.63	0.67	0.63	0.59	0.61	0.60	0.50	0.62	0.56

^*^p < 0.05; ^**^p < 0.01.

### 3.2 Satisfaction with sleep duration and life (Years I–IV)

**Figure 1:**
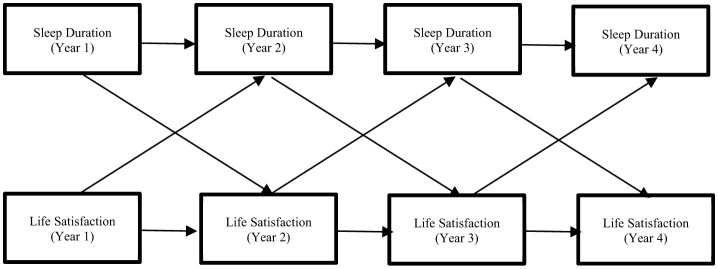
Sleep duration-life satisfaction ARCL model (Years I–VI).

To examine causality between sleep duration and life satisfaction, data from Years I–IV were utilized. Health conditions and gender from Year I were considered as control variables (Figure 1).

An invariance test was conducted to assess the relationship between sleep duration and life satisfaction. Firstly, the equivalence of autoregressive coefficients across different time points was examined. Secondly, the equality of cross-lagged coefficients was assessed. Thirdly, the equivalence of transposed variances was analyzed. Lastly, the equality of transposed covariances was tested. Further details are provided below:

*Model 1: A standard model without any constraints*
*Model 2: A model with homogeneity constraints imposed on the autoregressive coefficient of sleep duration*
*Model 3: A model derived from Model 2 with additional homogeneity constraints imposed on the autoregressive coefficient of life satisfaction*
*Model 4: A model derived from Model 3 with the imposition of homogeneity constraint on the cross-regression coefficient of sleep duration regarding life satisfaction*.
*Model 5: A model derived from Model 4 with homogeneity constraints applied to the cross-regression coefficient of sleep duration against life satisfaction*
*Model 6: A model derived from Model 5 with homogeneity constraints applied to the error covariance between sleep duration and life satisfaction*.

Table 3 presents the results of the goodness-of-fit testing for each model. Compared to Model 1, there was a decline in ΔCFI exceeding 0.01 in Model 2. Therefore, path-coefficient invariance was not supported (Cheung and Rensvold, 2002). Additionally, the RMSEA value exceeded 0.15, indicating that the conditions for invariance were not met (Chen, 2007).

**Table 3:** The goodness-of-fit of sleep duration-life satisfaction ARCL model.

**Model**	**Chi-square**	**df**	**NFI**	**CFI**	**RMSEA**	**ΔChi-square**	**df**	**ΔCFI**
Model 1	193.830^***^	13	0.949	0.951	0.076			
Model 2	342.455^***^	15	0.909	0.912	0.096	148.625	2	0.039
Model 3	352.092^***^	17	0.907	0.910	0.091	9.637	2	0.002
Model 4	354.589^***^	19	0.906	0.910	0.086	2.497	2	0
Model 5	395.798^***^	21	0.905	0.909	0.082	41.209	2	0.001
Model 6	366.587^***^	24	0.903	0.908	0.077	29.211	3	0.001

^***^p < 0.001.

### 3.3 Satisfaction with sleep duration and life (Years IV–VII)

**Figure 2:**
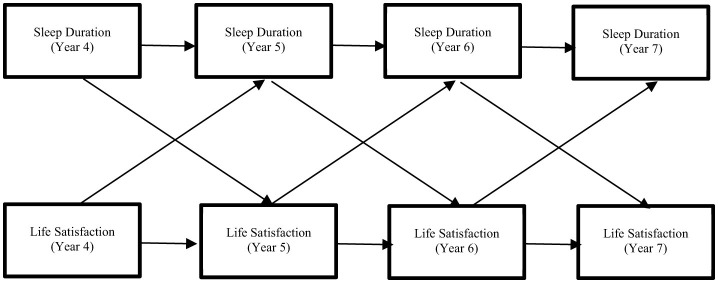
Sleep duration-life satisfaction ARCL model (Years VI–VII).

To analyze the second half of the 4th panel, data from Years IV–VII were utilized. Years IV and V correspond to 7th and 8th grades, respectively, while Years VI and VII represent 9th and 10th grades. For this analysis, health conditions and gender at Year IV were treated as control variables (Figure 2).

To analyze the relationship between sleep duration and life satisfaction, invariance testing was conducted, following the same procedure as in Years I–IV. Table 4 presents the results of the goodness-of-fit testing for each model. The findings indicated that ΔCFI did not decrease by more than 0.01 (Cheung and Rensvold, 2002), and the RMSEA did not exceed 0.015 (Chen, 2007). Hence, it is reasonable to conclude that invariance was achieved across all models. Consequently, Model 6 was selected as the final model.

**Table 4:** The goodness-of-fit of sleep duration-life satisfaction ARCL model.

**Model**	**Chi-square**	**df**	**NFI**	**CFI**	**RMSEA**	**ΔChi-square**	**Δdf**	**ΔCFI**
Model 1	345.323^***^	13	0.899	0.901	0.104			
Model 2	349.432^***^	15	0.898	0.901	0.097	4.109	2	0
Model 3	351.966^***^	17	0.897	0.901	0.091	2.534	2	0
Model 4	352.287^***^	19	0.897	0.901	0.086	0.321	2	0
Model 5	354.566^***^	21	0.896	0.901	0.082	2.279	2	0
Model 6	360.030^***^	24	0.895	0.900	0.077	5.464	3	0.001

^***^p < 0.001.

As indicated in Table 5, the CFI, NFI, and RMSEA values were 0.900, 0.895, and 0.077, respectively, reflecting the goodness of fit of the final model. Hence, it appears to be acceptable. A RMSEA of 0.077 suggests a reasonable fit when below 0.08 (Browne and Cudeck, 1993), thus supporting its acceptance.

**Table 5:** The final model's goodness-of-fit.

**Chi-square (df)**	***P***	**CFI**	**NFI**	**RMSEA**
360.030^***^ (24)	0.000	0.900	0.895	0.077

^***^p < 0.001.

The results of the analysis on the final model are presented in Table 6. Controlling for conditions before life satisfaction, an increase in sleep duration at the previous time points corresponded to improved life satisfaction at subsequent time points. Specifically, sleep duration during 7th grade exhibited a significant static influence on life satisfaction at 8th grade (β = 0.025, *p* < 0.03), while sleep duration during 8th grade statically affected life satisfaction during 9th grade (β = 0.026, *p* < 0.03). Moreover, sleep duration at 9th grade had a static influence on life satisfaction during 10th grade, with statistical significance (β = 0.027, *p* < 0.03). Additionally, life satisfaction did not show a significant influence on the sleep-duration cross-lagged coefficient (*p* > 0.10). In summary, as adolescents' sleep duration increased, their life satisfaction tended to improve.

**Table 6:** Path-coefficient estimates on sleep duration and life satisfaction.

**Variable**	**Unstandardized coefficient**	**S.E**.	**C.R**.	**Standardized coefficient**
Autoregressive coefficient on sleep duration < - sleep duration	0.401^***^	0.013	31.811	0.379/0.396/0.368
Autoregressive coefficient on life satisfaction < - satisfaction	0.423^***^	0.012	36.404	0.446/0.416/0.432
Cross-lagged coefficient on life satisfaction < - sleep duration	0.000^**^	0.000	2.195	0.025/0.026/0.027
Cross-lagged coefficient on sleep duration < - life satisfaction	1.640	1.273	1.288	0.017/0.016/0.015

^**^p < 0.05; ^***^p < 0.001.

Based on the aforementioned results, sleep duration during elementary school grades exhibits a significant influence on life satisfaction, beginning in the 7th grade. Consequently, it is crucial to prioritize sufficient sleep duration from the 7th grade onward to enhance life satisfaction.

## 4 Discussion

This study examined the causal relationship between adolescents' sleep duration and life satisfaction using the 4th-grade panel of the KCYPS. The findings revealed the following: Adolescents' sleep duration significantly and positively impacts life satisfaction from the 7th to 10th grades, whereas life satisfaction does not exert a significant effect on sleep duration. Thus, the study confirms the effects of sleep duration on life satisfaction. In essence, adjusting for sleep duration at previous time points may lead to improvements in life satisfaction at subsequent points in time.

Studies conducted in the West have shown that sufficient sleep and good sleep habits positively affect life satisfaction and happiness, significantly impacting emotional control abilities (e.g., Steptoe et al., 2008; Yoo et al., 2007). However, there are few studies focusing on Korean teenagers to determine whether sufficient sleep directly affects their happiness. Domestic research has shown that suicidal impulses increase with insufficient sleep (Park, 2015), but there is a lack of research on whether sufficient sleep directly improves happiness. Therefore, this study is significant as it emphasizes the importance of ensuring sufficient sleep to enhance the happiness of Korean adolescents.

Korean youth are under significant pressure from academic demands and the stress of college entrance exams. They face continuous exams twice every semester during middle school and high school, and college entrance exams add to this pressure. For teenagers striving to improve their grades with the belief that “studying is the only way to survive,” happiness is becoming increasingly elusive, while depression and stress dominate their lives. Additionally, they reduce their sleeping time to secure more time for studying. This study strongly emphasizes the need for changes in adolescents' lifestyles. To study effectively, it is important to prioritize securing more time for the brain to rest rather than simply increasing study hours. Research has shown that getting enough sleep positively impacts adolescents physically, mentally, and cognitively throughout their lives. Based on these findings, it is essential to change the environment in which adolescents are placed to ensure they get sufficient sleep, allowing them to change and improve their habits accordingly.

The significance of this study can be summarized as follows: First, utilizing data from the 4th-grade panel, the study confirmed the causality between sleep duration and life satisfaction employing the ARCL model. Particularly noteworthy is the division of data into two periods: 4th−7th grade and 7th−10th grade, considering the significant decline in adolescents' sleep duration upon entering junior high school. This approach allowed for an in-depth analysis of the relationship between sleep duration and life satisfaction across different stages of adolescence. Secondly, it was determined that the invariance of autoregressive cross-lag on sleep duration during the 4th−7th grade period was not confirmed while examining analysis conditions using the ARCL model. This suggests that the degree of change in sleep time across the 4th−5th, 5th−6th, and 6th−7th grade periods is not the same.

Drawing from the study results, future research topics were suggested as follows: As demonstrated in this study, there is a clear need to investigate the underlying causes of the significant causality between sleep duration and life satisfaction specifically during the 7th−10th grade period. In particular, it is essential to analyze these factors in conjunction with biological aspects such as hormonal changes induced by sleep duration and its impact on the emotional processing regions of the human brain. To achieve this, conducting research in collaboration with biotechnology and brain engineering experts is necessary. In addition, although this study did not determine the optimal amount of sleep for teenagers, it is necessary to conduct research that clearly establishes this. Applying different research analysis methods based on this data will likely provide sufficient insight into this issue. Thus, future studies should also aim to identify the appropriate sleep.

Obviously, this study has limitations. The first limitation is that we measured sleep duration based on the time the participants went to bed. However, the time of going to bed and the actual time falling asleep may differ, which can lead to inaccuracies in measuring actual sleep duration. The second limitation is that the life satisfaction items used in this study did not reflect concepts such as the adolescents' passion, will, or aspirations. The items used in this study were related to feelings of having no worries and feeling happy with life, which may limit the measurement of life satisfaction. Previous studies suggest that the concept of life satisfaction is multidimensional, encompassing various elements, as outlined in the Multidimensional Students' Life Satisfaction Scale (MSLSS). Therefore, it is expected that future research will use measurement items that include these various elements, leading to more meaningful results.

Finally, this study confirmed that adolescents' sleep duration declines as soon as they enter junior high school. This decrease appears to be closely related to an increase in depression among Korean adolescents. Therefore, it is necessary to create an environment that ensures sufficient sleep for children even after they enter junior high school. Parents need to understand the importance of sleep during adolescence, and school teachers should emphasize its importance to their students. Furthermore, schools need to take appropriate measures to ensure students can secure adequate sleep. It is necessary to review whether school start times are set too early and, in the case of high schools, to reconsider and adjust whether the end time for evening self-study sessions is too late. Parents also need to reflect on whether having their children attend private tutoring sessions late into the night contributes to sleep deprivation. They should evaluate whether they are prioritizing private education at the expense of their children's desired sleep schedule. Ensuring sufficient sleep time is essential for the happiness and wellbeing of adolescents. Protecting the right to sleep for adolescents is a must, and both parents and teachers should actively communicate the importance of sleep to adolescents, fostering understanding and support both at home and at school.

## Data Availability

Publicly available datasets were analyzed in this study. This data can be found at: https://www.nypi.re.kr/archive/eps.

## Footnotes

^1^This study is a revised and supplemented version of parts of the “Korean Children and Youth Panel Survey VIII: Data Analysis Report - The Positive Effects of Adolescents' Sleep Duration (Jung et al., 2017),” conducted by the National Youth Policy Institute (NYPI) in 2017.
